# Urban–Rural Partnership Framework to Enhance Food–Energy–Water Security in the Post-COVID-19 Era

**DOI:** 10.3390/ijerph182312493

**Published:** 2021-11-27

**Authors:** Priyanka Mitra, Rajib Shaw, Vibhas Sukhwani, Bijon Kumer Mitra, Md Abiar Rahman, Sameer Deshkar, Devesh Sharma

**Affiliations:** 1 Graduate School of Media and Governance, Keio University, Endo 5322, Fujisawa Shi 252-0882, Kanagawa, Japan; priyankamitra87@gmail.com (P.M.); vibhas@sfc.keio.ac.jp (V.S.); 2 Institute for Global Environmental Strategies (IGES), 2108-11 Kamiyamaguchi, Hayama 240-0115, Kanagawa, Japan; b-mitra@iges.or.jp; 3 Department of Agroforestry and Environment, Bangabandhu Sheikh Mujibur Rahman Agricultural University, Salna, Gazipur 1706, Bangladesh; abiar@bsmrau.edu.bd; 4 Department of Architecture and Planning, Visvesvaraya National Institute of Technology, South Ambazari Road, Nagpur 440010, Maharashtra, India; smdeshkar@arc.vnit.ac.in; 5 Department of Atmospheric Science, School of Earth Sciences, Central University of Rajasthan, NH-8, Bandar Sindri, Ajmer 305817, Rajasthan, India; deveshsharma@curaj.ac.in

**Keywords:** urban–rural partnership, food–energy–water security, global goals, sustainable development goals, COVID-19

## Abstract

Food, energy, and water (collectively referred to as ‘FEW’) security forms the key to human survival as well as socioeconomic development. However, the security of these basic resources is increasingly threatened due to growing demand. Beyond the widespread implications on public health, Coronavirus disease (COVID-19) has further raised additional challenges for FEW security, particularly for urban populations, as they mainly outsource their FEW demands from rural areas outside their physical boundaries. In light of that, this study reviews existing literature on FEW security to highlight the growing relevance of urban–rural linkages for realizing FEW security, especially against the backdrop of the COVID-19 pandemic. To achieve this, relevant research documents have been identified through Elsevier’s Scopus database and other sources (by applying search equations). The authors have accordingly underlined the necessity of shifting the conventional urban-centric approach to city region-centric development planning for the post-COVID-19 era. To this end, a framework has been suggested for translating physical urban–rural linkages to a partnership enhancing a collective response. The major elements of this framework are the conceptualization of national-level policies to support urban–rural linkages. The framework can play the role of a science–policy–action interface to redesign the FEW system in city regions.

## 1. Introduction

Rapid urbanization has today become one of the major sustainability challenges worldwide, particularly in developing countries [1]. By 2018, urban areas were already the habitat of around 55% of the world’s total population, a proportion due to reach 68% by 2050 [2]. Correspondingly, urban areas have emerged as the demand centers of natural resources such as food, energy, and water [3,4]. At the same time, this trend in rapid urbanization is resulting in inequality, unsustainability, polarization, and divergence in terms of development and social inclusion between urban and rural areas [5]. Due to the growing concentration of economic activities and services, cities are becoming the preferred destinations for rural migrants, wishing to pursue employment opportunities and improved quality of life [6]. Unplanned urbanization and rural-to-urban migration create various social, environmental, and sustainability challenges. A business-as-usual development approach prioritizes mainly economic and social dimensions, with environmental impacts often sidelined [7]. However, to realize sustainable development, development planning needs to be conducted through an integrated lens of economic growth, social development, and environmental conservation [8]. In the context of sustainable urbanization, managing economic prosperity and protecting the natural resources in rural regions can ensure food, energy, and water (collective referred to as ‘FEW’) security for urban areas [9]. Hence, the interlinkage of urban and rural has come into focus for sustainable and resilient development. A sustainable development approach, in the context of developing countries, needs to be viewed as a means of optimizing interactions between various underlying factors including poverty, climate change, rapid urbanization, and food insecurity that can make or mar socioeconomic development and environmental conservation, rather than being regarded as an ideal development pathway.

Typically, urban–rural linkages are referred to as the spatial flows of people, capital, goods, services, sectorial and financial flows, and information between rural and urban areas [10,11]. Thus, the umbrella of urban–rural linkages covers a broad variety of themes within the domain of urban and territorial planning, such as strengthening small and intermediate towns. The associated functions of social links, economic dynamics, and environmental synergies maintain the interdependencies between urban and rural areas. Encouraging an urban and rural partnership in a local context is important for realizing a transformation towards sustainable development. The importance of urban–rural partnership is, accordingly, being acknowledged in global and regional development agendas in European countries, and in development policies worldwide. For instance, Japan’s 5th Basic Environment Plan recognized the necessity of urban–rural linkage for economic revitalization, and for a low carbon, and resilient society, introducing the Circulating and Ecological Sphere (CES) concept [12].

As urban populations heavily rely on rural areas for their FEW needs [13,14], the importance of urban–rural partnership has been highlighted by several studies [15,16,17]. Since population growth increases food demand in urban areas, the strengthening of urban–rural connections has become a determinant for food security and nutrition [18]. In parallel, the urban–rural partnership is also gaining attention, with cities declaring their commitment to the race for a net-zero emission society and capturing the potential of solar energy resources that are available in rural areas. Several cities in Europe, Japan, and other regions are now establishing a collaboration to achieve 100% renewable targets by 2050 [19,20].

Coronavirus disease 2019 (COVID-19), caused by the novel coronavirus, was first detected in December 2019, and the World Health Organization (WHO) declared the coronavirus outbreak a pandemic on 11 March 2020. In response to the pandemic, various responses have been imposed by the national and local governments that brought changes in urban–rural flows, including people’s movement, food supply chain, services, etc. The widespread implications of the pandemic on human societies, beyond the implications on public health, have raised additional questions about the conventional concepts of urban development and resilience, particularly in developing countries. Taking due account of supply chain disruptions, it has been realized that urban–rural interlinkages must be carefully considered in the responses to COVID-19, and recovery strategies and actions, as well as the localization of the FEW security, should be emphasized to reboot the global economy.

A great deal of existing literature focuses on FEW sectors independently [21,22,23], however few studies have discussed the relationship between urban–rural partnership and the FEW nexus [24,25]. Some studies related to the FEW nexus and COVID-19 have also been published [26,27,28]. However, the importance of urban–rural partnerships to strengthen the FEW nexus for the post COVID-19 era has not yet been discussed. In light of that, this paper advances the current understanding of urban–rural linkages, framing them within the integrated management of FEW for the post COVID-19 era.

Mainly based on a literature review, the three key objectives of this study are: (1) To review existing literature on urban–rural linkages and their relevance for FEW security; (2) To understand the effects of the COVID-19 pandemic on FEW security; (3) To suggest a feasible policy level framework to translate urban–rural linkages to partnership.

Overall, this paper comprises six sections. Section 2 establishes a theoretical foundation (on urban–rural linkages and FEW nexus) for the readers to better comprehend this research. Section 3 describes the adopted research methods, while Section 4 provides an overview of the research findings. Within the broader discussion of study findings, Section 5 discusses the suggested urban–rural partnership framework, and some key conclusions and research limitations are summarized in Section 6.

## 2. Literature Review

### 2.1. Significance of Urban–Rural Linkages

**Figure 1:**
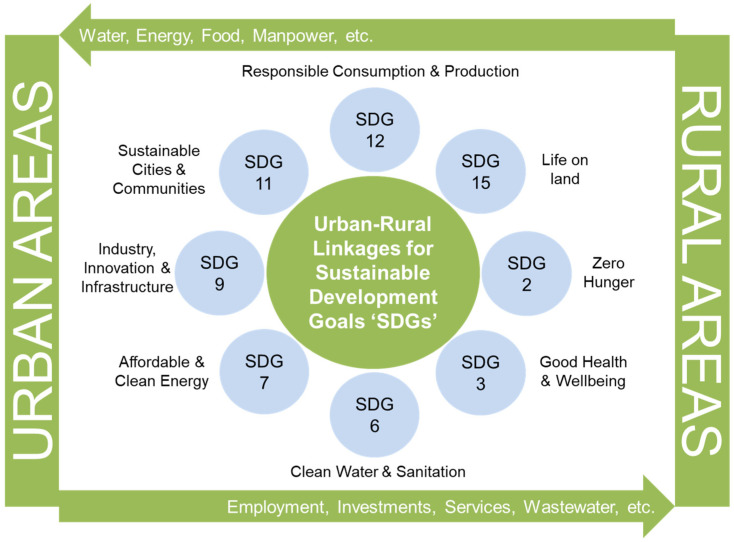
Conceptual diagram of urban–rural linkage with sustainable development goals (SDGs).

Urban and rural regions exist as interdependent entities connected through various spatial and sectoral flows (refer to Figure 1). Rural regions serve as centers for key resources such as food, water, energy, and labor, which are crucial for urban regions. Similarly, urban regions provide opportunities for rural dwellers, creating markets for agricultural products, and becoming sources of temporary employment and shelter. Urban–rural linkages can be defined as the direct (and two-way) flow of resources between geographically dispersed urban and rural areas [10,29,30]. Enhancing the continuity and connection between urban and rural regions is crucial for reducing poverty, achieving a satisfactory level of access to and management of resources, and at the same time maintaining the ecological and cultural diversity that is essential for regional resilience. On the other hand, unplanned and rapid urbanization is attributed to the loss of agricultural land, wetlands, and forest [25,31].

### 2.2. Urban–Rural Linkage for Achieving Global Agenda

The importance of linkages between urban and rural areas is recognized in global frameworks such as the 2030 Agenda, with its sustainable development goals (SDGs, refer to Figure 1) and the Sendai Framework for Disaster Risk Reduction, and was first acknowledged in the Vancouver Action Plan (Habitat I) [32]. The commission on Human Settlements in 1999 also appealed to consider the urban–rural interdependence in the implementation of the UN-Habitat program [33]. In 2003, the UN-Habitat emphasized urban–rural linkages in the publication “Urban-Rural Linkages Approach to Sustainable Development”. At the United Nations conference in 2012, member states also committed “to work towards improving the quality of human settlements, including the living and working conditions of both urban and rural dwellers in the context of poverty eradication so that all people have access to basic services, housing, and mobility” [34]. The significance of urban–rural linkages has also been described further in the New Urban Agenda in 2016, where member states committed to supporting “the role of small and intermediate cities and towns in enhancing food security and nutrition systems, providing access to sustainable, affordable, adequate, resilient and safe housing, infrastructure and services, and facilitate effective trade links across the urban-rural continuum” [35]. This agenda has encouraged the implementation of sustainable urban and territorial planning to promote interactions among urban, peri-urban, and rural areas, in both developing and developed countries.

SDG 11—“Sustainable cities and communities” emphasizes the importance of national urban policies and regional development plans for positive economic, social, and environmental links between urban and rural areas. It calls for sustainable urbanization incorporating participatory approaches and the integration of climate change and disaster resilience into development policies and plans at all levels of settlement planning. Urban–rural interlinkages are also critical for achieving other SDGs, including SDG 2 (food), SDG 6 (water and sanitation), SDG 7 (energy), and SDG 15 (land) (as highlighted in Figure 1 earlier).

Urban–rural linkages can play an important role in mitigating the risks of both natural and man-made disasters. The Sendai Framework for Disaster Risk Reduction 2015–2030 also encourages an upgrade of knowledge on disaster risk for all possible aspects of exposure, properties of vulnerability and hazard, strengthening of disaster risk governance, and accountability for disaster risk management [36]. Urban–rural linkages can support modifying the situation of disasters in rural areas and can help minimize the vulnerability of rural residents [37]. Moreover, many development agencies, such as the Organization for Economic Cooperation and Development [38], the Department for International Development [39], and the World Bank [40] are now placing urban–rural linkage in their investment projects, thereby recognizing poverty mitigation and achieving broader equality.

#### 2.2.1. Urban–Rural Partnership to Implement Regional Development Plan (EU Rurban Program)

Agreed by the European Parliament in 2010, RURBAN refers to the actions for sustainable urban–rural development and partnerships, managed by the European Commission. The European Union (EU) has given due importance to urban–rural linkages while working together with other countries and regions on the understanding that stronger urban–rural linkage is more beneficial in terms of more efficient land use and planning, efficient services, and sustainable natural resource management. Herein, the urban–rural partnership is increasingly being considered as a tool for regional development to replace the conventional policies of discrete urban and rural development. Starting from the second half of the 1990s, the EU has also raised awareness on urban–rural partnership through specific research programs such as Study Programme on European Spatial Planning (SPESP), European Spatial Planning Observation Network (ESPON), and the 6th Research Framework Program. Consequently, several national level initiatives have emerged to promote urban–rural partnerships in European countries such as Germany, the UK, France, and Spain.

It has been realized that urban–rural partnerships cannot be established without engaging local actors. This is because the process and outcomes are influenced by local and regional conditions. The EU has highlighted the urban–rural partnership as a specific form of governance for integrated territorial development. Primarily, such partnerships are set on a sub-regional level that is supported by a regional and national–legal and financial framework. Nevertheless, the EU provided major value to promote urban–rural partnerships depending on the community objective of economic, social, and territorial cohesion. Urban–rural partnerships were put forward in a rather new and challenging dimension, distributing the policy and funding of European cohesion and rural development to member states and regions. The EU framework requires specific methods to incorporate urban–rural issues into future programs. However, the EU administrative bodies were reluctant to include new strategic issues or governance approaches to complement urban–rural partnerships using a certain tool for experimentation, innovation, capitalization, and developing policy.

#### 2.2.2. Urban–Rural Linkage in the Basic Environment Plan of Japan

Against the backdrop of recent global policy agreements such as the 2030 Agenda for Sustainable Development, the 5th Basic Environment Plan was approved by the Ministry of Environment of Japan (MOEJ) in 2018. The two main ideas outlined in this plan are respect for planetary boundaries and urgent achievement of the SDGs [41]. Therefore, MOEJ introduced the CES concept in its plan for a sustainable transition. This concept is an integrated policy approach that includes three principal elements: (1) a low-carbon society, (2) resource circulation, and (3) living in harmony with nature. These elements were proposed in *Becoming a Leading Environmental Nation in the 21st Century—Japan’s Strategy for a Sustainable Society* in 2007. For this, a framework was established within the CES to promote the interaction and cooperation of the three elements. The concept of a circular economy and low-carbon society is built on the ideas of reducing waste, producing renewable sources of energy, and employing ecosystem services without any damage which ultimately optimizes human activities and minimizes their impact on nature [42]. The CES encourages spatial linkages to establish advanced methods for sustainability and integrated responses at a local scale, which are in contrast to existing approaches. There are four approaches to execute the CES: (1) urban–rural linkages, (2) ecosystem-based solutions, (3) decarbonization, and (4) resource circulation.

The CES concept motivates the formation of intricate and more sustainable urban–rural linkages based on the current flows of food, goods, people, capital, waste, natural resources, and renewable energy. These strengthened linkages are intended to carry potential revised chains of ecological production and consumption in such a way that cities and towns can meet their primary resource demands within regional boundaries and become self-sufficient for energy and food while reducing waste [43].

### 2.3. Urban–Rural Linkage for Optimization of FEW Nexus

The FEW nexus refers to the tight interconnection between food, energy, and water systems. Furthermore, the security of each of the independent resources is reliant upon the realization of the overall FEW security. Nevertheless, demand for FEW in urban areas is met through both local in-boundary and, to a larger extent, transboundary production [44], and this rising urban demand has far-reaching environmental impacts, from in-boundary to outside of urban area boundaries. Accordingly, there is a need to optimize the production and supply of FEW resources to urban areas, at both city in-boundary and transboundary scales. A potential approach to realize this could be the evaluation of trade-offs and co-benefits among environmental effect categories, which can be inferred through water and energy footprints [44]. Water footprints inform on water removal from watersheds, risk of water scarcity in the operation of industries or power plants, and risks to crop production. This type of evaluation of trade-offs enables sufficient measures to be taken at a suitable level in four key categories to gain water security: (1) changes in FEW demand, (2) in-boundary versus transboundary FEW supply relocation, (3) intervention of in-boundary production system, and (4) intervention in trans-boundary production along with cross-sectoral food, energy, and water interactions. Execution of these measures requires interventions in supply–demand governance, integrated energy planning, integrated use planning, technological interventions, lifestyle change, and ways to engage citizens in the decision-making process at the urban–rural functional region level.

## 3. Research Methods

For reviewing the state-of-the-art scientific literature related to urban–rural partnership and FEW security, two major sources are considered. The first is peer-reviewed literature identified through the Elsevier’s Scopus data: The search was performed with keywords ’Food, Energy, Water, Security, Urban, Rural’ in the ‘ALL’ category, and a total of 39 literature were retrieved initially (on 22 March 2021). Another search was performed on the same date with the keywords ‘Food, Energy, Water, Security, COVID-19′ in the ‘ALL’ category, and a total of 38 documents were found. After a manual screening of literature titles, abstracts, and sometimes full papers, the relevant documents were extracted for review. The second source is grey literature identified through other sources: Beyond the Scopus search for peer-reviewed literature, other online and offline sources were also considered to identify relevant (and up-to-date) research documents, such as government documents and reports, international organization documents and reports, conference proceedings, and newspapers. Similar keywords to those used for Scopus were also applied to other search engines (including Google Scholar), thereby identifying relevant documents to gain a better understanding of the subject and on the impacts of the COVD-19 pandemic.

## 4. Growing Relevance of Urban–Rural Linkage for FEW Security

### 4.1. Urban–Rural Linkage for Food Security

Rapid urbanization is reshaping the land-use pattern in functional urban–rural regions, which increases the challenges of achieving the global goal for zero hunger (SDG-2). Urbanization plays a significant role in increasing the demand for agricultural products in terms of both the growing population and dietary changes, which may increase risks to food security both for urban and rural populations [45]. Rural agricultural production is the major source of food for people living in contiguous urban–rural areas [46]. Loose interconnections between urban and rural areas have a negative effect on food security and nutrition for vulnerable people in cities and rural communities [47,48]. Strengthening the urban–rural linkage is one of the keys to mitigating the risk of food insecurity. Furthermore, strengthened urban–rural linkages can support inclusive economic growth by connecting rural agricultural production with urban markets, creating food supply chain-related, non-farm business and job opportunities in rural, peri-urban, and urban areas, and promoting urban technical support and investments in farms located in rural areas. Changes in the dietary choices of the urban population also influence crop cultivation. To mitigate these concerns and to strengthen urban–rural linkages, especially against the backdrop of pandemic emergencies, there is a need for city–region level responses, several examples of which are highlighted in Table 1.

The impact on food systems due to the COVID-19 pandemic calls attention to the opportunities that can be captured by connecting local production and consumption. Cities and wider regions need to have an action plan for helping resilient food systems, ensuring that: (1) the food supply chain is diversified and resilient to future shocks, (2) food access is maintained, and (3) mitigation is in place for vulnerable food system actors, which include small-scale producers, migrant labors, low-income, and neglected groups.

### 4.2. Urban–Rural Linkage and Energy Security

Huge investment is required for electricity production, and these are highlighted when moving towards decarbonized cities and industrial activity and managing investment for renewable options. Renewable technologies provide an edge over fossil fuel options as they produce little or no air pollution and can be utilized instantly. Renewable technologies are also economically helpful. The cost of electricity that solar photovoltaics and offshore wind generate is competitive with fossil power, resulting in costs being cut by 25–40% between 2018 and 2023 [52]. Renewable energy has become an essential part of the solution towards a net-zero emission world. Today, there are more than 100 cities globally that are mainly powered by renewable energy, most of which are in Africa, Europe, North America, and South America. Among these 100 renewable cities, only Inje in the Republic of Korea is located in Asia. Many other cities across the world have set a target to achieve a 100% renewable energy supply by 2050. Achieving 100% renewable energy within city territory is challenging considering the limited available space and lack of natural resources. Therefore, many cities (several examples shown in Table 2) are now building partnerships with neighboring or far distant rural areas for achieving a 100% renewable supply [53].

Among the listed cities in Table 2, Yokohama City in Japan and Frankfurt City in Germany have set targets to achieve 100% RE by 2050 through a combination of energy use efficiency improvement and increased energy supply from renewable sources. However, both these cities have limited resources within the city boundaries to ensure 100% RE generation in the city region. For example, it is estimated that Yokohama needs to receive 19.1 billion KWh in energy supply from renewable sources to achieve the ambitious carbon neutral target by 2050. However, estimates have revealed that only 8% of the total energy demand can be produced within the city boundary. Therefore, Yokohama needs to secure the remaining 92% from outside of the city area through collaboration with other cities, towns, and villages, which have abundant RE resources. Yokohama has accordingly signed agreements with 12 local governments in the Tohoku region with abundant RE sources. Under these agreements, the partner cities, towns, and villages supply RE to Yokohama City. This collaboration has helped create a circulating and ecological sphere to generate a positive economic cycle, including RE and environmental value. Similarly, the city of Frankfort has established collaboration with stakeholders in the city area, harnessing RE potential beyond the city boundary that can cover 184% of its power needs by 2050 [54]. This cooperation opens up new opportunities to convert cities and regions from energy consumers to RE prosumers, thereby creating value.

### 4.3. Urban–Rural Linkage and Water Security

Due to rapid urbanization, the demand for water for urban populations is projected to increase by 50–80% by 2050 [55]. To meet the growing urban water demand in the face of declining freshwater availability, the most common response is water reallocation from rural to urban regions. The increase in such trends implies that there are growing conflicts between cities and their surrounding rural areas, particularly in the global south [56,57]. A survey identified at least eight important issues that need urban–rural cooperation, including water allocation, water quality, flood control, water allocation, wastewater treatment, physical accessibility to a water source, water storage, provision of water services, and ownership of water [58]. In light of that, urban–rural collaborations can offer a win-win solution for water security at the regional level. Several good examples of urban–rural partnerships for water management and security have been identified in different places (Table 3). These include water use efficiency improvement in agriculture (e.g., Southern California in the US, Reus in Spain), improving groundwater recharge (e.g., Kumamoto in Japan), managing water source forest (Kanagawa in Japan), and promoting environment-friendly organic agricultural practices (Munich in Germany). Introducing incentive mechanisms can enhance urban–rural cooperation on water management and water security. Urban–rural cooperation can also generate multiple benefits for both sides, including water security for urban populations and increased incomes for rural people, as well as creating new job and business opportunities.

### 4.4. Impact of COVID-19 on FEW Nexus

**Figure 2:**
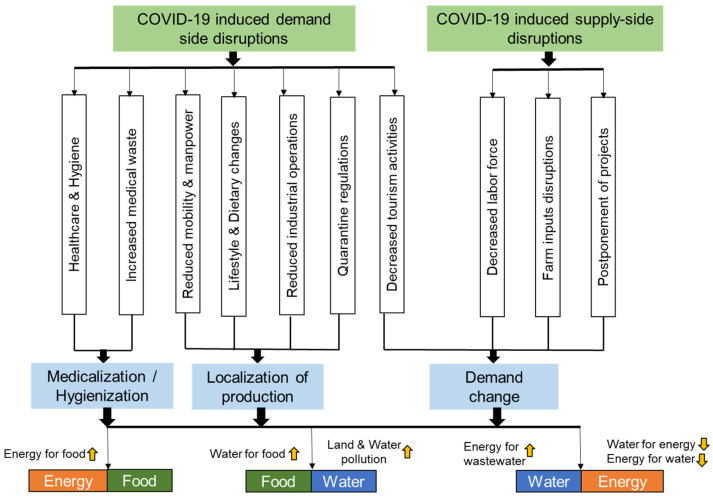
Implications of COVID-19 on the FEW nexus (image source: modified from [26]).

Since 2020, the world has been facing the devastating effects of the COVID-19 pandemic, which has had a direct impact on FEW security [26,27,28] in terms of both demand-side and supply-side disruptions (overview presented in Figure 2). For instance, nearly 2.1 billion people worldwide already lacked access to safely managed drinking water [59], and so frequent handwashing has emerged as a constrained resource when trying to follow the World Health Organization’s advisory on hygiene. In the case of demand-side disruptions, increased hygiene habits and medicalization has led to increased medical waste generation, which may have significant impacts on soil or water pollution if not properly treated. The treatment of additional medical waste or wastewater also requires more energy supply [60,61,62,63]. Water is further considered as the key component to fight against COVID-19 globally, as countries that lack chemical-based disinfectant depend more on a water source [64,65]. The water ecosystem is also greatly affected by biomedical waste, and this is hampering water supply sources and ultimately harming the environment [66]. In Bangladesh, at least 14,500 tons of waste from healthcare was generated across the country due to COVID-19 in April 2020 [67]. India also experienced a 46% increase in COVID-19-related biomedical waste generation in between April and May 2021, with many municipalities of India recording a sharp increase (up to 25%) in domestic water consumption (e.g., Kozhikode in Kerala and Ahmedabad in Gujarat) [68].

Food security is also under stress due to widespread disruptions along the supply chain, as well as impacts on agricultural production due to mobility restrictions [69,70]. Demand-side disruptions include reduced mobility, dietary changes, reduction in industrial operations, and quarantine regulations, and these force people to take a local production approach. This implies that the FEW nexus at the local level should be assessed and optimized to avoid tradeoffs. If local production is to increase, then more water and energy will be required from local sources.

Demand-side disruptions such as a reduction in everyday mobility for work or social events results in low energy demand. Decreased leisure activities are also responsible for lowering energy demands. Lockdowns are expected to further disrupt energy demands significantly as primary energy consumption decreased [71]. It has been reported in some European countries that energy demand fell during the first wave of COVID-19 [72]. The pandemic also affected tourism, also resulting in lower demands for energy along with food and water. On the other hand, quarantine regulations may result in higher electricity and water requirements. These kinds of measures influence the overall FEW resource consumption profile.

The cross-linking perspective of COVID-19 and FEW nexus is also considered to prioritize links to reduce the complexity of disruptions. Increased hygiene activities and biomedical waste has an effect on the two sub-nexuses: the food–water sub-nexus due to pollution of the land and, consequently, the water sources, and the energy-water nexus due to increasing demand for water and energy for wastewater treatment. On the other hand, disruptions due to decreased mobility and dietary changes during quarantine are forcing basic supplies to be produced locally [73,74,75]. This impact points to crosslinks between COVID-19 and both the food–water sub-nexus and the energy–food sub-nexus, as enhanced local food policies require water and energy. There are also other disruptions that will result in changes in the demand for water or energy resources. For example, staying at home increases electricity and water demand [76]. With the increased energy demand, energy management systems are increased. Due to this kind of instability, it is difficult to assume what the exact COVID-19-related changes are in terms of water use for electricity supply.

## 5. Discussion

### 5.1. Strengthening Urban–Rural Linkages for FEW Security in Post-COVID-19 Era

From this review of existing literature, we can conclude that the importance of FEW security is increasingly being recognized at policy and governance levels, although at varying levels and sectoral considerations. It is also being recognized that cities are increasingly reliant on their surrounding rural areas for FEW supplies [77]. For instance, in terms of energy security, it is underlined in Section 4.2. that most cities cannot achieve net-zero emission within their city boundary. While nearly 60% of the world’s population lives in cities (that occupy only 2% of land area) [35], in-boundary FEW production is constrained due to high density and limited space. On the other hand, peri-urban and rural areas often lack the infrastructure they require, including human, financial, and technical resources to ensure an optimal use of regional natural resources.

As highlighted in Section 2, the importance of urban–rural linkages and FEW resource security (such as water for all, clean energy, and food security) has, accordingly, been acknowledged on global agendas, in regional programs, and for national development strategies. As discussed in Section 4.1, urban–rural linkage plays a very important role for food security, particularly in times of emergency. Enhancing local production and local consumption can thus improve resilience for food supply systems against the various externalities such as disruptions during pandemics. In response to the food supply risk due to the COVID-19 pandemic, a city–region food system approach has been promoted in different parts of the world (see Section 4.1). This approach clearly acknowledged the critical role of urban–rural linkages in mitigating food insecurity during emergencies.

Global leaders are working together, aiming for a net-zero emission world by mid-century, and, as such, a huge expansion of renewable energy (RE) would play a vital role in achieving this goal. As cities account for 70% of the world’s energy consumption, actions in and by cities have received special attention for achieving important global goals and national development targets. In fact, more than 700 cities have made a wide range of commitments for achieving carbon neutral or net-zero emission targets by 2050 [78]. Achieving these commitments requires radical decarbonization measures such as achieving 100% RE, which, in many cases, go beyond city boundaries. Cities need to explore collaboration with surrounding rural areas to utilize the maximum potential of available decarbonization options that can be the engine to achieve net-zero emission. As discussed in Section 4.2, several cities in developed countries have realized that they must collaborate with rural areas if they want to achieve their 100% RE energy targets. This can generate a positive economic cycle, linking RE and environmental values. Urban–rural partnerships for the expansion of RE can also bring new job opportunities. Grid-connected renewables are mainly located outside of cities, which can create new job opportunities in rural areas. It is estimated that the RE sector will create nearly 300,000 new job opportunities in India by 2022 [79]. In particular, RE power plants can create permanent job opportunities for those people who migrated to rural are due to the pandemic [80].

While poor water management in terms of both water use efficiency and water environment conservation are some causes of increased water insecurity worldwide, Section 4.3 shows how collective urban–rural actions can help to improve water management in different regions. Cities need to secure reliable and quality water supply for their growing populations, as well as for fueling economic activities. Offering economic incentives can enhance improvements in water use efficiency and water environment conservations in rural communities, as observed in the cities of San Diego in US, Kumamoto in Japan, Reus in Spain, and Munich in Germany (refer to Table 3).

Broadly, FEW security is inherently inter-linked and inter-dependent. Overlooking the nexus that exists for FEW resources management in rural areas not only aggravates the risk to FEW sectors in those areas, but it also creates threats to FEW security for urban populations. Furthermore, the COVID-19 pandemic also places additional concerns on the FEW nexus, as responses to the pandemic have direct impacts on FEW due to disruption on both supply and demand side, as discussed in Section 4.4. In the post-COVID-19-era, more emphasis should be put on the localization of FEW security and resilience to mitigate such risks in the future. There is also likely to be an increased demand for local ecosystem services related to FEW due to intensive economic activities and demography changes. This implies that the dynamics of the FEW nexus will also change along with promotion of localization. Therefore, the local-level FEW nexus, particularly in the urban–rural continuum, is likely to also gain more attention in the post-COVID-19 era.

Here lies an opportunity to create a win–win relationship between urban and rural areas toward a self-reliant and resilient region. Cities can take advantage of the abandoned natural resources available in rural areas to meet the growing demand for FEW for their population. On the other hand, a city can offer necessary support for better management of the FEW nexus in rural areas in order to ensure an efficient and optimum use of resources for higher productivity. This can also generate multiple benefits for rural populations, including inclusive economic growth and new job opportunities.

### 5.2. Framework for Translating Urban–Rural Linkage to Partnership

In the post-COVID-19 era, building urban–rural partnership is essential to formulate a sustainable recovery plan for FEW systems and to redesign these systems. This can be achieved by acknowledging the interdependence of urban and rural areas, thereby optimizing the FEW nexus.

Conventional urban and rural governance is designed for the management of issues and challenges within a specific area’s own spatial boundaries only, and underestimates the value of surrounding landscapes for their resilience and sustainable development. Ignorance of urban–rural linkages in the national legislation and policies is also one of the main reasons for the disconnected management of FEW resources. In recent years, urban–rural linkages have been strengthened in some developed countries or regions, with national governments and regional authorities formulating strong supporting policies. For example, urban–rural linkages have been promoted in some of Japan’s major government policy documents such as its Basic Environmental Plan. Furthermore, several supporting schemes have been launched to promote urban–rural linkages. As discussed in Section 2, urban–rural partnerships have also been promoted by the European Union with various supporting schemes, and many European countries have established good practices in urban–rural linkages that can serve as guidance for developing countries. This implies that national governments should take proactive steps to promote urban–rural partnership by formulating appropriate legislation, strategic guidelines, and relevant policies, as well as by offering technical and financial assistance.

**Figure 3:**
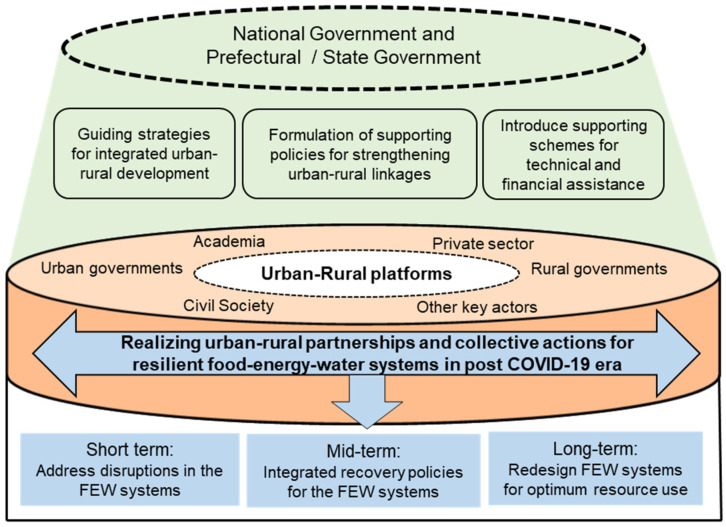
Framework of urban–rural partnership for capturing multiple benefits (image source: authors).

These national-level interventions are expected to trigger cooperation initiatives between urban and rural governments. Upscaling the sustainability of urban–rural cooperation requires a platform that brings together all key stakeholders from urban–rural regions to ensure horizontal coordination and to formulate a common vision and integrated actions for a sustainable FEW system. This platform can play the role of a science–policy–action interface by connecting people, skills, and money from the city region. Strengthened urban–rural partnerships will lead to a collective response to address disruptions in the system, formulation of integrated recovery policies, and a redesign of FEW systems through the optimum use of local resources, skills, and finance. A strong urban–rural partnership will not only enhance the resilience of the city region, but will also contribute to the localization of national and global goals. Figure 3 illustrates a suggested framework for enhancing urban–rural partnerships.

## 6. Conclusions

This study highlights the importance of strengthening urban–rural linkage for the collective security of FEW in the post-COVID-19 era. Based mainly on a review of the existing literature, this study develops a precise understanding of the importance of urban–rural linkages and sets out a city-region perspective for FEW security. It also synthesizes the impact of COVID-19 measures on the FEW nexus, for disruption to both demand and supply, discussing how the pandemic and various countermeasures have altered the relationship across the FEW nexus. A framework on how to translate urban–rural linkages into partnerships has also been suggested to manage FEW systems in a collective manner. The important elements of the proposed framework are the formulation of national-level policies to support urban–rural linkages, the provision of guidance and technical and financial assistance through national schemes, and the engagement of multistakeholder platforms at a city-region level. Multistakeholder platforms can play a role as science–policy–action interfaces that enhance the collective response, sustainable recovery plans, and the redesign of the food, energy, and water nexus in an urban region.

To meet the increasing demand for FEW by rapidly growing populations is challenging, particularly for urban areas that mostly rely on supply from outside of city boundaries. Hence, urban–rural linkages should be properly acknowledged for enhancing the security of FEW resources. This study found that existing literature highlights the application of urban–rural linkage for FEW security, however focuses on a single or dual resource security issue. While FEW security is interlinked, this study argues for collectively managing the FEW nexus both on the supply side (rural areas) and demand side (urban areas) through strengthening urban–rural linkages.

The authors acknowledge that this research is subject to certain limitations, as it is mainly based on a review of existing literature. Although the study findings are expected to remain relevant for the long term, the situation surrounding the COVID-19 pandemic is changing constantly, and a growing number of studies are further highlighting good examples of FEW security from around the world. The future scope of this research accordingly entails case-specific studies at a local level, wherein the applicability of the proposed framework for translating urban–rural linkages into partnerships can be tested.

## Figures and Tables

**Table 1:** Examples of city–region food system approaches in response to pandemics.

City	City Region Food System Approach	Source
Antananarivo, Madagascar	Mapping of food flow, regulating product quantities along with discovering the importance of each actor in the food chain.	[49]
Medellín, Colombia	In response to the COVID-19 crisis, the territorial position and the city region food systems notion have been incorporated in the city’s food and nutrition security programs.	[50]
Colombo, Sri Lanka	CRFS has been introduced to ensure communities can easily access food by any alternative supply chain linkages by synchronizing with multi-stakeholders across administrative boundaries.	[51]

**Table 2:** Urban–rural partnership for achieving renewable energy target of selected cities.

City	Country	Vision	Urban–Rural Partnership
Barcelona	Spain	An energy-independent city through achieving 100% renewable energy (RE) by 2050	In light of the limited resources and opportunities at the city level, the municipality coordinates with the wider metropolitan area to achieve the 100% RE vision by 2050.
Frankfurt	Germany	100% RE by 2050 50% energy savings 25% RE generation within the city territory 25% RE produced in the region (metropolitan area)	It is difficult for the city of Frankfurt to achieve its “100% renewable” target on its own. It needs resources from the metropolitan area and even regional level for wind power and biomass.
Frederikshavn	Denmark	100% RE by 2030	The objective of 100% RE cannot be achieved without transforming the resources to the energy available in the surrounding area of Frederikshavn. Biomass is considered as an opportunity to boost agriculture while it develops as an energy supplier. Off-shore wind power is also tackled through DONG Energy.
Geneva	Switzerland	100% RE by 2050	To explore the locally available wood biomass, the municipality contributed to establishing a local industry, where benefits come through municipal forestry and from a partnership with the Geneva Association of private forest owners to ensure that the selling price of wood biomass is fair. This partnership ensures private owners’ sustainable and free-of-charge management of forests. It promotes job opportunities in the local area.
Yokohama	Japan	Achieve carbon neutral by 2050 8% RE produce within city 92% RE supplied from outside of the city	Yokohama has concluded agreements on RE with 12 municipalities including Kuji City, Ninohe City, Kuzumaki Town, Fudai Village, Karumai Town, Noda Village, Kunohe Village, Hirono Town and Ichinohe Town, Aizuwakamatsu City, and Koriyama City, which have abundant renewable resources based on CES concept.

**Table 3:** Urban–rural cooperation for water management and water security.

Location	Issues	Mechanism of Collaboration	Benefits
Southern California, US	Water stress and vulnerability to drought	San Diego city-initiated agreement to give compensation to the farmers for water conservation	Nearly 100 million cubic meters (MCM) were saved by farmers and sent to the city. It is target is 237 MCM by 2021
Reus, Spain	Water allocation problem between cities and agriculture	A water market mechanism was introduced by the irrigation subscriber association that includes the Reus city, other municipalities, and small rural landowners. The water right is distributed based on fixed price	- Reduced urban water demand - Increased water use efficiency in agriculture - Revenues are used to finance dams and other infrastructures
Kumamoto, Japan	Kumamoto City, which completely relies on groundwater resources faced groundwater level depletion	Incentivizing paddy field owners for groundwater recharge	- Increased groundwater recharge that improved water security for the city - Improved income of paddy field owners
Kanagawa, Japan	Water quality of major water source rivers of prefectural water is being affected due to poor management of water source forest	Introduced conservation and restoration of water source environment through taxation and this revenue is used for supporting management water catchment areas in the upstream	- Improved quality of river water quality - Support for local forest businesses - Creates jobs in forest management sector
Munich, Germany	The city water supply source, the Mangfall valley, experienced nitrate and pesticide pollution due to intensive agricultural practices	The municipal water utility introduced a voluntary payment scheme to promote organic farming	- Improved water quality (nitrate concentration reduced to 7 mg/L) - Reduced water treatment cost - Large market for organic farming in Germany
